# PNA Clamping in Nucleic Acid Amplification Protocols to Detect Single Nucleotide Mutations Related to Cancer

**DOI:** 10.3390/molecules25040786

**Published:** 2020-02-12

**Authors:** Munira F. Fouz, Daniel H. Appella

**Affiliations:** Laboratory of Bioorganic Chemistry, National Institute of Diabetes and Digestive and Kidney Diseases, National Institutes of Health, Department of Health and Human Services, Bethesda, MD 20892, USA; fmuniracmu@gmail.com

**Keywords:** peptide nucleic acids (PNAs), single-nucleotide polymorphism (SNP), polymerase chain reaction (PCR), cancer.

## Abstract

This review describes the application of peptide nucleic acids (PNAs) as clamps that prevent nucleic acid amplification of wild-type DNA so that DNA with mutations may be observed. These methods are useful to detect single-nucleotide polymorphisms (SNPs) in cases where there is a small amount of mutated DNA relative to the amount of normal (unmutated/wild-type) DNA. Detecting SNPs arising from mutated DNA can be useful to diagnose various genetic diseases, and is especially important in cancer diagnostics for early detection, proper diagnosis, and monitoring of disease progression. Most examples use PNA clamps to inhibit PCR amplification of wild-type DNA to identify the presence of mutated DNA associated with various types of cancer.

## 1. Introduction

Single nucleotide changes occurring within a normal (often called wild-type) DNA sequence may be associated with different diseases, and in particular, the development or progression of various cancers [1]. When such changes occur, the normal nucleotide may be replaced with one of the three other possible nucleotides. These replacements are called single-nucleotide polymorphisms (SNPs). Many SNPs occur at an approximate frequency of 1 out of 1000 bases in the human genome [2], and SNPs associated with diseases may be important signals of the presence and severity of an illness. 

Certain SNPs in genes can be used to detect various diseases, including: solid tumors [3,4,5], childhood leukemia [6,7], metabolic disorders [8,9], diabetes [10], and gout [11]. SNPs may also signal patient-to-patient differences associated with responses to drug treatments [12,13]. In the area of cancer diagnostics, SNPs in genes such as KRAS [14,15], EGFR [16,17], p53 [18], FLT3 [19], or KIT [20] are associated with lung cancer, colorectal tumors, and blood-based cancers. Detection of these mutations signals the presence of tumor cells, which is important for early diagnosis as well as for gauging the effectiveness of ongoing therapy to treat tumors. 

Detection of SNPs in clinical samples is challenging, as the diagnostic assay used must be very sensitive and very specific. Considering the heterogeneous distribution of tumors, SNPs associated with cancers are typically present in small quantities relative to normal, unmutated, wild-type DNA in clinical samples. Despite their low level of abundance, the presence of certain SNPs may determine the response of patients to selected therapeutic regimens and drugs [21,22,23,24]. Therefore, it is crucial to refine the reliability and sensitivity of SNP detection methods so that specific personalized treatments can be more accurate [25]. 

Numerous methods exist to detect SNPs. Some existing methods are polymerase chain reaction (PCR) restriction fragment length polymorphism mapping (PCR-RFLP), allele-specific PCR (AS-PCR), allele-specific hydrolysis or dual hybridization probes, high resolution melting analysis (HRMA), amplification refractory mutation system (ARMS), dual priming oligonucleotides (DPO), TaqMan allelic discrimination assay, pyrosequencing, next generation sequencing (NGS), IntPlex, BEAMing, and droplet digital PCR (dPCR) [26,27,28,29,30,31,32,33,34,35]. Most of these methods can detect DNA with a single mutation when they are present at only 1% to 5% relative to the amount of wild-type DNA in a sample. Methods such as IntPlex, BEAMing, and dPCR can give sensitivities up to 0.0005% [36]. However, there are limitations to such nonconventional methods when applied in the clinic. The IntPlex method requires individual DNA-specific primers for each specific type of mutant, and only one mutant can be detected in a single tube, although other variants for the same SNP may be present [37,38]. The BEAMing technique requires tumor DNA to be amplified, followed by processing an emulsion, fluorescent tagging, and analysis of beads using flow cytometry [39]. The droplet dPCR method is still relatively challenging to implement as it can be expensive, labor intensive, and requires specialized emulsion instrumentation [40,41].

Promising methods for SNP detection include polymerase chain reaction (PCR)-based assays in addition to isothermal amplification methods which use a clamp that is designed specifically to block the amplification of wild-type DNA while allowing amplification of a much smaller amount of mutated DNA that contains a SNP [42]. In this review, we report on the use of peptide nucleic acids (PNA)-based clamps with a specific emphasis on their application to the identification of various cancers.

## 2. The Concept of PCR Clamping via PNA

**Figure 1:**
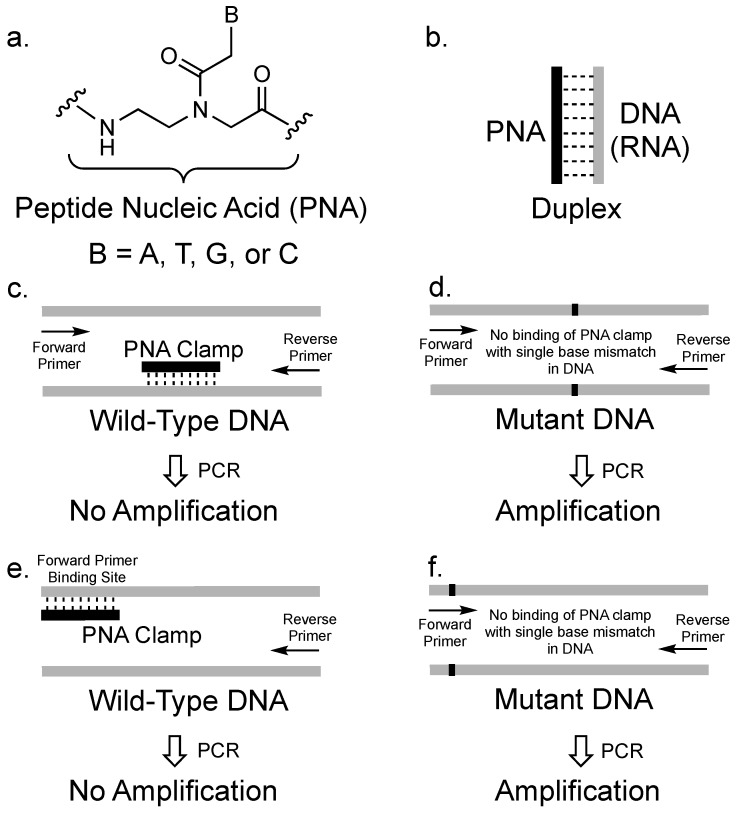
(**a**) Chemical structure of the peptide nucleic acid (PNA) backbone. (**b**) Representation of PNA forming a duplex with complementary DNA or RNA. (**c**) Inhibition of PCR amplification of wild-type DNA by elongation arrest due to the strong binding of a PNA clamp to the DNA. (**d**) Without the PNA clamp binding, mutant DNA amplification by PCR can proceed. (**e**) Inhibition of PCR amplification of wild-type DNA by PNA binding to the forward primer binding site. (**f**) Without the PNA clamp binding, the forward primer can bind to the mutant DNA sequence and amplification by PCR proceeds.

PNAs were first designed in the laboratory of Peter Nielsen and Ole Buchardt [43]. In contrast to natural nucleic acids, PNAs consist of nucleobases attached to amide-linked N-(2-amino-ethyl)-glycine units instead of a sugar phosphate backbone (Figure 1a). PNAs are achiral and uncharged molecules that are chemically stable and resistant to enzymatic degradation. Furthermore, PNAs are capable of sequence-specific recognition of DNA and RNA sequences following the typical Watson-Crick hydrogen bonding patterns (Figure 1b). The resulting hybrid PNA-nucleic acid duplexes exhibit high thermal stability. Since PNAs were first developed, they have attracted attention due to their potential utility in diagnostic and pharmaceutical applications [44,45,46,47,48,49,50].

Ørum et al. first introduced PNA as a clamp in real time PCR to specifically block amplification of a wild-type DNA so that a mutated DNA that differs by a single nucleotide could be selectively amplified [51] (Figure 1c,d). In PCR, a target nucleic acid sequence (called a template) is amplified by a DNA polymerase enzyme. The template is typically a DNA sequence, and the most commonly used enzyme is the thermostable *Taq* DNA polymerase. For PCR to proceed, short, synthetic DNA sequences (called primers) that are 15-40 bases long must be designed to bind to the ends of both strands of the DNA templates. Primers are necessary, as the polymerase must bind to duplex DNA to begin elongation. Once the polymerase binds to the DNA duplex consisting of the primer bound to the template, deoxyribonucleoside triphosphates (dNTPs) are enzymatically added to the primers to make a complimentary copy based on the template DNA. This process is iteratively repeated to achieve exponential amplification of the original DNA template [52]. 

Although functional as binders of nucleic acids, PNAs are chemically different from DNA such that they behave as clamps to inhibit PCR amplification. Specifically, PNAs cannot function as primers for DNA polymerase as they are intrinsically resistant to the DNA-specific enzymatic activity normally associated with *Taq* DNA polymerase. Therefore, the PNA can be designed to bind to a DNA template and inhibit elongation of DNA by halting the polymerase activity (Figure 1c). Elongation arrest is one mechanism by which PNAs may act as a clamp to inhibit PCR amplification. PNA/DNA interactions are commonly 1 °C per base pair more stable than the corresponding DNA/DNA duplex. When PNA binds to a mismatched DNA sequence, the resulting duplex is more destabilized by the mismatch than the corresponding DNA/DNA duplex of the same sequence [53]. In addition to elongation arrest, the thermal stability and sequence specificity of PNA binding to DNA allows properly designed PNAs to competitively exclude DNA primers from binding to a DNA template (Figure 1e,f), providing another mechanism by which PNA clamps can inhibit PCR amplification. Therefore, PNAs can be used to prevent PCR amplification of a target DNA sequence. However, given the single-nucleotide recognition sensitivity of PNAs, a DNA with a slightly different sequence may not be clamped by the PNA and may be therefore selectively amplified. To function as a clamp, a PNA does not have to completely inhibit amplification of a target DNA template. According to Orum et al., when a template is the target of a clamp, the effect of incomplete blocking on amplification of the clamped DNA can be mathematically calculated [54]. For example, in the case of a PNA clamp designed to block amplification based on competitive primer binding that allows about 10% of the target DNA to be amplified, approximately 10,000 copies of the clamped DNA would be present after 30 PCR cycles. This amount should be much smaller compared to any unclamped DNA, which should theoretically have around 2 billion copies after 30 cycles.

## 3. PCR Clamping via PNA to Detect Mutated DNA in Cancer

The ability to analyze and monitor the occurrence of mutations in specific cancer-associated genes (called oncogenes) is important for the early detection of cancer and also to determine the effectiveness of chemotherapy treatments [55]. Numerous studies have shown that mutations in the KRAS oncogene may play a key role in the development of different cancers. KRAS encodes for a 21-kDa GTP-binding protein that influences cell growth and differentiation. Mutations in KRAS may lock a cell into a state of uncontrolled growth, ultimately resulting in growth of a cancerous tumor. In patients with metastatic colorectal cancer (mCRC), analysis of mutations in KRAS codons 12 and 13 is commonly performed before starting treatment with cetuximab or panitumumab, which are antibody-based therapeutic medicines that target the epidermal growth factor receptor (EGFR) [36]. Both antibodies bind to EGFR and block binding of the natural ligand, as well as prevent receptor dimerization and activation of the related cellular signaling pathways [56]. However, cetuximab and panitumumab are only approximately 10% and 30% effective in patients, respectively [57]. Clinical studies have demonstrated that patients with mCRC who have wild-type (non-mutated) KRAS respond better to therapy than those who have mutations in KRAS. Therefore, detecting KRAS mutations is important to identify which patients would respond best to therapy. The challenge, however, is that the level of DNA associated with a mutant form of KRAS may be very low relative to the amount of wild-type KRAS DNA, even in a cancer patient. [58]. 

Thiede et al. [59] provided the first example of PNA clamping to detect mutations in KRAS. The KRAS mutations most commonly known to promote cancer are in a 4–5 base pair sequence of DNA in codons 12 and 13 of the gene. A PNA clamp specific to the wild-type KRAS gene suppressed its amplification to allow selective amplification of the less abundant mutations that occur in codons 12 and 13 of KRAS. This strategy was tested on six of the twelve possible KRAS mutations derived from different tumors. The identity of the mutation was confirmed by sequence analysis of the amplified mutant KRAS DNA. To test the sensitivity of the assay, mixtures of wild-type and mutated DNA templates were analyzed and it was determined that mutant DNA could be detected at levels as low as 0.5% relative to the amount of wild-type DNA present in the same sample. In contrast, sequence analysis of the same mixture of wild-type and mutated DNA amplified without the PNA clamp failed to detect the mutation. Therefore, the enrichment of the mutated DNA in the PNA-clamped amplification was important for proper detection and identification. 

Since the initial work on KRAS, others have used PNA clamps for the purpose of cancer diagnostics. Chen et al. [60] developed a technique using PNA clamps and fluorescent probes that bind amplified mutated DNA to detect KRAS mutations in bile samples obtained from 116 patients with biliary obstruction. They compared their technique with restriction fragment length polymorphism (RFLP) analysis. After DNA extraction, PCR and RFLP were used to detect KRAS mutations, which were confirmed by sequence analysis. Using the PNA-clamped PCR assay, DNA with mutations in KRAS were detected under 1 h at a level of 0.03% relative to the amount of wild-type DNA. In contrast, RFLP analysis detected the mutated DNA at a level of 1% at best and also needed about 2 days for the analysis. In another report that examined mutations in KRAS, Taback et al. [61] used a PNA-clamped PCR assay specific for KRAS mutations to assess sentinel lymph nodes (SLN) for occult CRC micro-metastases. In their study, the PCR protocol with a PNA clamp was optimized to detect 0.05% of mutated DNA in the presence of wild-type DNA and the method was used to examine mutations in 72 patients with CRC. In addition, Däbritz et al. [62] employed PNA clamped real time PCR with specific hybridization probes and melting curve analysis to examine common mutations in codon 12 of KRAS in tissue and plasma samples of patients with pancreatic cancer. The sensitivity was optimized to detect 0.001% of mutated DNA in the presence of wild-type DNA.

PNA clamps to prevent PCR amplification of wild-type DNA can be applied with various types of instrumentation. In addition to their use as PCR clamps, PNAs may also be used as probes to signal the detection of mutated DNA after PCR amplification. Luo et al. [63] have developed a method to detect trace amounts of mutant KRAS in a single step by using a PNA clamp to suppress wild-type KRAS during capillary PCR. Interestingly, they also used a PNA labeled with a fluorescent dye to serve as a sensor probe to differentiate all 12 possible mutations from the wild-type by a melting temperature (*T*_m_) shift of 9 to 16 °C. Mutated DNA could be detected at levels as low as 0.01% relative to wild-type DNA, and the method successfully detected mutated DNA in 19 samples out of a group of 24 serum samples from patients with pancreatic cancer. Although the results of that study were impressive, the requirement to use a LightCycler (Roche Diagnostics, Mannheim, Germany) PCR instrument to perform the assay may reduce the versatility of this method in broader clinical settings.

While *Taq* DNA polymerase is commonly used for PCR, the enzyme’s error rate during replication can lower the accuracy of any diagnostic that relies on the polymerase. In an assay that uses PNA clamps with PCR, the PNA is supposed to suppress amplification of wild-type DNA while allowing mutant DNA to be amplified. If there are polymerase errors during the amplification that happen to occur in the same region of DNA where the PNA clamp binds, then these errors will not be clamped by the PNA as there will be a mismatch between the sequences. Therefore, the error will likely be amplified along with the mutant DNA, and this may lead to incorrect analyses. Gilje et al. [64] have shown that there can be problems in PNA clamped PCR due to the low fidelity of *Taq* DNA polymerase. By switching to a high-fidelity polymerase (Phusion HS) that is about 50 times more accurate than *Taq* DNA polymerase, the sensitivity to detect mutant KRAS DNA increased approximately 10-fold. Mutant KRAS DNA could be detected at levels as low as 0.005% relative to wild-type DNA. Therefore, replication errors due to the fidelity of *Taq* polymerase should be considered as a potential source of error in PNA-clamped PCR assays that may limit the sensitivity.

PNA clamps are remarkably compatible with many different strategies for nucleic acid amplification, including isothermal methods. Araki et al. [65] evaluated a technique called PNA-clamp smart amplification process version 2 (SmartAmp2) to detect KRAS mutations in patient samples. SmartAmp2 uses specially designed sets of primers to target six distinctly different sequences of a template DNA containing a specific mutation, achieving selective amplification of the mutant sequence via a self-priming mechanism. When successful, a mutant DNA sequence may be detected in one step within 30 min under isothermal conditions. Using a PNA clamp designed to suppress the wild-type DNA sequence of KRAS, amplification of mutated DNA in codon 12 of KRAS was achieved. Samples from 172 patients with lung adenocarcinoma were analyzed by the PNA clamped SmartAmp2 to determine how well mutations in codon 12 of KRAS could be detected compared to other methods. The method detected mutations in 31 of the samples, which was better than other PCR methods without the PNA clamp. 

PNA clamps have been successfully used with asymmetric PCR in which one DNA strand is preferentially amplified. Oh et al. [66] demonstrated that PNA clamped PCR can be used in combination with asymmetric primers for amplification followed by melting curve analysis that relies on binding of unlabeled, C-6 amino-modified DNA detection probes to the amplified DNA. Asymmetric PCR was used to generate higher amounts of the antisense DNA strand which is the DNA to which the unlabeled detection probes must bind. A nice feature of this approach is that different mutations may be identified based solely on differences in the melting temperatures of the probes when bound to amplified DNA. Mutant KRAS DNA can be detected at levels of about 0.1% relative to wild-type DNA, which is not as sensitive as other methods. Nevertheless, the simplicity of the protocol, the unlabeled detection probes, and the ease of data analysis provides for an assay that may be highly useful.

The backbone of the PNA clamp in every method described so far in this review has consisted of the simple polyamide backbone depicted in Figure 1a. In contrast, Kim et al. [67] developed a unique approach to detect and identify multiple KRAS mutations using chemically modified PNAs both as clamps and as detection probes. According to their approach, one PNA would serve as a clamp to suppress amplification of wild-type DNA. A separate detection PNA would target a mutant DNA and signal both its quantity and identity. The detection probe PNA was designed with a fluorophore and quencher at opposite ends so that it would fluoresce upon binding to its complementary DNA. The challenge with implementing this approach stems from the requirement to design both the clamp and detection PNAs with sequences that are almost completely complementary to each other. When two PNAs are complementary, they can bind to each other with very high affinity instead of binding to DNA. Therefore, the clamp and detection PNAs had to be modified to prevent them from binding to each other, and the binding affinities of the detection PNAs to different mutant DNAs had to be unique for each mutant sequence (as measured by *T*_m_ values). 

**Figure 2:**
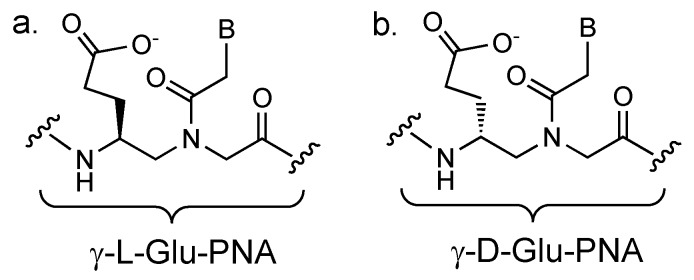
(**a**) Chemical structure of the γ-L-Glu-PNA backbone that binds complementary DNA. (**b**) Chemical structure of the γ-D-Glu-PNA backbone that does not bind complementary DNA.

To adjust the binding properties of the PNAs, sidechains may be introduced into the γ position of the PNA backbone to either increase or decrease binding affinities to complementary sequences [68]. The sidechain at the γ position is derived from either an L- or D-amino acid. PNAs with γ sidechains derived from an L-amino acid tend to increase the binding affinity to complementary DNA, while sidechains derived from a D-amino acid tend to decrease the binding affinity. In their study, Kim et al. [67] determined that a sidechain from either L- or D-glutamic acid (Glu) proved to be the most useful for adjusting binding affinities of the PNAs (Figure 2). For the clamp, a γ-PNA derived from L-Glu was designed to slightly increase the binding affinity to the wild-type DNA. The detection probes, in contrast, were γ-PNA derived from D-Glu and they bound with slightly weaker affinities than the clamp to their mutant DNA target sequences. The ability to alter the binding affinities of the different PNAs was important for the success of the study. Furthermore, the use of opposite chirality in the two types of PNA prevented them from binding to each other. This assay was applied to the detection and identification of six different mutant DNAs of KRAS (at the same gene region) with a 1% sensitivity relative to wild-type DNA.

The technology to sequence DNA has rapidly advanced over the past several years, with the term next-generation sequencing (NGS) describing high-throughput sequencing technology that has resulted in faster and more efficient collection of genomic data. Despite these advances, the errors associated with NGS data can range from 0.1 to 15%, and therefore the detection of mutations present at a low level compared to unmutated DNA can be problematic [69]. In an attempt to improve the ability of NGS to detect mutations, Rakhit et al. [70] developed a PNA clamp to bind wild-type KRAS during the PCR amplification stage of the NGS library preparation. To test the method, they used circulating-free DNA (cfDNA) derived from a patient with advanced non-small cell lung cancer in which a KRAS mutation was present at 3.2% relative to unmutated DNA. The patient’s DNA was amplified by PCR both in the presence and absence of the PNA clamp, followed by NGS analysis of both sources of DNA. The authors nicely demonstrate that the PNA-clamped samples showed an increase in the number of mutant reads and that the associated mutation frequency relative to wild-type DNA in the NGS analysis also increased. Despite their success, the authors point out that the use of the PNA clamp in the NGS workflow makes the resulting data only qualitative in nature, and they point out that the PNA clamp could have off-target effects that negatively impact the detection of other regions of the DNA. The use of PNA clamps to assist NGS analysis is clearly possible, but more work in this area is necessary to determine whether their application is truly beneficial.

While PNA clamps have been mostly applied to the analysis of KRAS mutations, there are some other cancer targets to which PNAs have been applied as clamps to detect mutations. The protein p53 is a tumor suppressor that is typically activated in response to cell damage to instruct the damaged cell to stop growing or instruct the cell to undergo programmed cell death (apoptosis). Mutations in the DNA encoding p53 are commonly seen in many different cancers [18]. One of the initial studies to apply PNA clamping to PCR for the detection of mutations in p53 DNA was by Behn and Schuermann [71]. A PNA clamp was used to lower the amount of wild-type p53 DNA amplified by PCR so the subsequent analysis by single-strand conformational polymorphism (SSCP) could achieve detection of mutated DNA at a level of 0.5% relative to wild-type DNA. They validated their assay using samples from patients with lung cancer. The same authors further improved their method using nested PCR amplification after the initial PCR amplification, and compared the results both with and without the PNA clamp [72]. Without the clamp, mutated DNA could be identified at a level of 5% relative to wild-type DNA, and with the clamp this amount was lowered to 0.1%. The assay was validated using samples from patients with lung cancer. The examples cited above relied on a PNA clamp that bound directly to wild-type p53 DNA at locations where the mutations occur. Myal et al. [73] also used a PNA clamp to detect p53 mutations, although with a slightly different strategy. In their work, they used PNA clamps to compete with PCR primers binding to p53 DNA for detection of mutated p53 DNA down to the level of 0.05% of mutated DNA relative to wild-type DNA.

PNA clamps have also been applied to the detection of mutated DNA associated with epidermal growth factor receptor (EGFR), which is a tyrosine kinase. Mutations in EGFR may determine the responsiveness of some cancers to treatment with different chemotherapies, and PNA clamps have been examined to help analyze mutations in the DNA encoding this receptor. Specifically, EGFR mutations may impact treatment with gefitinib, which is a tyrosine kinase inhibitor used to treat lung cancers [74]. It has been observed that gefitinib is effective in some patients yet ineffective in others, and some of these differences are linked to mutations in EGFR. Patients with tumors that have certain EGFR mutations may show improved responses to gefitinib. However, other EGFR mutations confer tumor resistance to gefitinib. Therefore, identifying mutations in EGFR may greatly assist treatment for several different cancers. 

In this regard, Nagai et al. [75] developed a detection system for EGFR mutations using a combination of PNA clamps to suppress amplification of wild-type DNA and locked nucleic acids (LNA) with fluorescent groups as probes to signal the presence of mutant DNA. LNAs are another class of nucleic acid analogs that bind to complementary DNA sequences with high thermal stability and with very good sequence specificity [76], and LNA was used so that it would not interfere with PNA binding to the wild-type DNA. This system successfully detected mutated DNA at a level of 0.1% relative to wild-type DNA, and it was used to test samples from patients with non-small cell lung cancer. 

One of the most successful patient studies performed using a PNA clamp was reported by Kim et al. [77]. In their study, samples from 240 patients with metastatic non-squamous non-small cell lung cancer (NSCLC) were examined for the presence of mutant EGFR DNA using both direct DNA sequencing as well as PNA clamped PCR to suppress amplification of wild-type EGFR DNA followed by sequencing. Mutations were detected in 83 of the samples using the PNA clamp protocol, while only 63 samples with mutations were identified by direct sequencing alone. When searching for known mutations, the PNA clamped protocol was demonstrably better at amplifying the signal for detection. The PNA clamp in this study came from a kit called PNAClamp Mutation Detection Kit (Panagene, Daejeon, South Korea).

In a related study, Yam et al. [78] relied on a PNA clamp to both identify mutant EGFR in patients with NSCLC and also to continue to test several of the same patients in follow-up tests after treatment. The detection assay relied on a PNA clamp to suppress amplification of wild-type EGFR DNA in a type of asymmetric PCR that produced mostly single stranded products. Analysis was then performed using a microarray to bind the single stranded product, followed by incorporation of a fluorescent nucleotide in subsequent primer extension and finally analysis of the microarray by a laser to determine the mutations present. The technique is very sensitive, with the ability to identify mutated DNA at a level of 0.1% relative to wild-type DNA. Drug resistant mutations can occur in patients taking tyrosine kinase inhibitors to treat NSCLC. Using their technique, eleven different types of EGFR drug-resistant mutations were identified in plasma-DNA from the patients, and 21 patients were followed for up to 18 months. Patients who responded to therapy had undetectable levels of mutated DNA, while drug resistant mutations were detected in some of the patients who failed to respond to the therapy. 

The use of cfDNA to identify EGFR mutations has the potential to improve clinical tests as it is easier to obtain compared to other methods of sample collection (such as a biopsy). Considerable additional research needs to be performed to validate this method using such samples to predict clinical outcomes. For this reason, Kim et al. [79] used a PNA-clamped PCR method to study EGFR mutations in cfDNA isolated from plasma samples from 60 patients with NSCLC who had shown a partial response to treatment with gefitinib. The authors used the same PNAClamp Mutation Detection Kit described previously. While the assay to detect mutant DNA was sensitive, the patient samples showed only a low level of mutated DNA from EGFR. The authors conclude that the use of cfDNA in cancer diagnostics requires additional study. To improve the clinical utility of cfDNA, Han et al. [80] used a PNA clamp in conjunction with melting curve analysis in PCR to follow both EGFR and KRAS mutations in the plasma of patients with NSCLC. The PNA clamp was part of a kit called PANAMutyper^TM^ (Panagene, Daejeon, South Korea). Using this method, they were able to discriminate between mutated and wild-type DNA by melting temperature differences with sensitivity around 0.1%–0.01%. Their results showed that the technique can be used to monitor cfDNA in patients. However, they also conclude that additional work must be performed before cfDNA is used more widely to make clinical decisions. 

## 4. Conclusions and Future Perspectives 

The sensitivity of PNA clamp-based PCR assays is extremely good when the PCR assay uses target-specific probes that bind to mutant sequences. Table 1 summarizes the different oncogenes mentioned in this review. In particular, fluorescent probes with strong binding affinity to target sequences, such as LNAs, can significantly enhance the limits of detection of mutant sequences. However, some oncogenes, such as KRAS, have many different mutations, and this situation may require making several different fluorescent probes to detect every different mutant. The application of PNA clamps in several clinical articles cited in this review, as well as the use of commercial kits featuring PNA clamps, highlight the promising development of using PNA clamps for clinical diagnostics related to cancer.

## Figures and Tables

**Table 1:** List of mutant oncogenes detected with PNA clamped nucleic acid amplification. Accompanying methods used for detection are described in column 2. Limits of detection are listed in column 3 (N/A means the information was Not Available). Length of the PNA used is listed in column 4 (kit refers to a PNA that was part of a kit and the PNA length was not described). References are listed in column 5.

Oncogene	Method Used in Combination with PNA Clamped PCR	Mutated DNA Detected in Presence of Wild-Type DNA	PNA Sequence Length- Number of Nucleobases	Refs
KRAS	DNA Sequencing	0.5%	15	[59]
	Fluorescent Probes	0.03%	17	[60]
	Melting Curve Analysis	0.05%, 0.001%	15,17	[61,62]
	Fluorescent PNA Sensor with LightCycler	0.01%	17	[63]
	High Fidelity DNA Polymerase	0.005%	17	[64]
	SmartAmp2	1%	17	[65]
	Asymmetric PCR	0.1%	17	[66]
	Modified PNA Detection Probes	1%	17	[67]
	Next Generation Sequencing	N/A	6	[70]
p53	PCR-SSCP	N/A,0.1%,0.05%	15,15,15	[71,72,73]
EGFR	PNA+LNA	0.1%	14-18	[75]
	DNA Sequencing	0.1%	Kit	[77]
	Fluorescent Melting Curve Analysis	0.1%,N/A,0.01%	Kit	[78,79,80]
